# Pancreatic Tumorigenesis: Precursors, Genetic Risk Factors and Screening

**DOI:** 10.3390/curroncol29110686

**Published:** 2022-11-15

**Authors:** Mohamed Badheeb, Adham Abdelrahim, Abdullah Esmail, Godsfavour Umoru, Karen Abboud, Ebtesam Al-Najjar, Ghaith Rasheed, Mohammed Alkhulaifawi, Ala Abudayyeh, Maen Abdelrahim

**Affiliations:** 1 Internal Medicine Department, College of Medicine, Hadhramout University, Mukalla 50512, Yemen; 2 Endoprothic Center, Hochwald Hospital, 60488 Frankfurt, Germany; 3 Section of GI Oncology, Department of Medical Oncology, Houston Methodist Cancer Center, Houston, TX 77030, USA; 4 Department of Pharmacy, Houston Methodist Hospital, Houston, TX 77030, USA; 5 Faculty of Medicine and Health Sciences, University of Science and Technology, Sana’a 15201, Yemen; 6 Faculty of Medicine, The Hashemite University, Zarqa 13133, Jordan; 7 Kharkov National Medical University, Kharkiv 6100, Ukraine; 8 Section of Nephrology, Division of Internal Medicine, The University of Texas MD Anderson Cancer Center, Houston, TX 77030, USA; 9 Weill Cornell Medical College, New York, NY 14853, USA; 10 Cockrell Center for Advanced Therapeutic Phase I Program, Houston Methodist Research Institute, Houston, TX 77030, USA

**Keywords:** pancreatic cancer, tumorigenesis, screening, cancer, ctDNA

## Abstract

Pancreatic cancer (PC) is a highly malignant and aggressive tumor. Despite medical advancement, the silent nature of PC results in only 20% of all cases considered resectable at the time of diagnosis. It is projected to become the second leading cause in 2030. Most pancreatic cancer cases are diagnosed in the advanced stages. Such cases are typically unresectable and are associated with a 5-year survival of less than 10%. Although there is no guideline consensus regarding recommendations for screening for pancreatic cancer, early detection has been associated with better outcomes. In addition to continued utilization of imaging and conventional tumor markers, clinicians should be aware of novel testing modalities that may be effective for early detection of pancreatic cancer in individuals with high-risk factors. The pathogenesis of PC is not well understood; however, various modifiable and non-modifiable factors have been implicated in pancreatic oncogenesis. PC detection in the earlier stages is associated with better outcomes; nevertheless, most oncological societies do not recommend universal screening as it may result in a high false-positive rate. Therefore, targeted screening for high-risk individuals represents a reasonable option. In this review, we aimed to summarize the pathogenesis, genetic risk factors, high-risk population, and screening modalities for PC.

## 1. Introduction

Pancreatic cancer (PC) is a highly aggressive malignant tumor and is the seventh highest cause of cancer-related mortality worldwide, the fourth most common in the United States (US), and projected to become the second leading cause of cancer-related mortality by 2030 [1]. Each year in the United States, more than 60,000 patients are diagnosed with PC. Unfortunately, most of these patients die from cancer-related consequences [2,3]. PC is more prevalent among those aged older than 45 years old, with a peak incidence among those aged 65 and 75 years old for males and females, respectively [4]. In addition, gender, ethnicity, and racial factors may influence PC incidence; for instance, males, blacks, and African Americans have a higher incidence than other groups [5,6]. The tumorigenesis of PC is not well understood, with various associated genetic mutations and derangements causing genomic instability and subsequent tumor progression. In addition, PC appears to be an end result of precursor lesions that evolve with the acquisition of somatic mutations in a step-wise fashion [7]. PC is frequently used to indicate pancreatic ductal adenocarcinoma, which accounts for 85% of pancreatic malignancies [8].

Despite various medical therapeutical advancements, the overall survival of PC has barely improved [9]. In nodal-positive PC, the 5-year survival rate is 8–10% [10]. Although the 5-year survival rate may reach up to 30% in the absence of nodal involvement, 70% of patients present with nodal involvement upon diagnosis [11,12,13]. In addition, less than 20% of cases are considered resectable at presentation, and the majority of patients present with metastatic or advanced disease at diagnosis. However, the widespread use of imaging has resulted in increased detection of stage IA PC. Fortunately, the 5-year overall survival associated with this early-stage PC has significantly increased from 45% to 84% [14].

Previous studies have shown that PC screening may be associated with improved outcomes including reduced mortality and a prolonged median cancer-specific survival rate in high-risk individuals [15]. Most oncological societies, however, do not support universal screening for PC due to high false-positive rates. In addition to increased medical expenditures, more invasive testing may be performed, which can be hazardous and carry emotional distress for the patients and their families. Nevertheless, it would not be unreasonable to target high-risk individuals who can benefit from such testing [16]. Family history and specific associated conditions may be utilized to identify patients who are high risk and may potentially benefit from screening for pancreatic cancer. Different screening modalities have been used in practice for PC screening including imaging, serological, and pathological testing. Liquid biopsy has arisen as a non-invasive method to detect PC, including circulating tumor DNA (ctDNA), circulating tumor cells (CTCs) and microRNAs (miRNAs) [17,18]. Unfortunately, there is no reliable serological marker for the early recognition of PC. In this review, we aim to discuss the pathogenesis of PC, including tumorigenesis, precursor lesions, and associated genetic risk factors. In addition, we discuss different screening approaches and their modalities.

## 2. Pancreatic Tumorigenesis

PC is often used to describe pancreatic ductal adenocarcinoma (PDAC), which accounts for 85% of pancreatic malignancies. However, pancreatic neoplasms can be further classified based on origin as exocrine or endocrine pancreatic neoplasms, accounting for 95% and 5%, respectively [8,19].

### 2.1. Pathophysiology of Pancreatic Cancer

The complexity of PC arises from the various mutations associated with it. Although ATM, BRCA1/2, and CDKN2A are linked to PC initiation and progression, KRAS mutation appears to be the most prevalent [20].

Mutated RAS genes, such as HRAS, NRAS, and KRAS, have also been observed in 10–30% of human cancers. However, the latter represents 86% of RAS family mutations [21,22,23]. KRAS is activated upon binding to a guanosine triphosphate (GTP) molecule forming KRAS-GTP, a process promoted via guanine nucleotide exchange factors (GEFs). Upon activation, KRAS propagates downstream signaling involving mitogen-activated protein (MAP) kinase, phosphoinositide-3- kinase (PI3K), and Ras-like guanine nucleotide exchange factor (RALGEF) pathways, which leads to an enhanced cellular division, growth, differentiation, and survival [24,25,26,27]. Under normal physiologic circumstances, KRAS is switched to an off state with the aid of its intrinsic GTPase, which hydrolyzes GTP into guanosine diphosphate (GDP) and renders KRAS inactivated, a process that is enabled via GTPase-activating proteins (GAPs) [28,29,30,31]. Oncogenic KRAS in pancreatic acinar cells has also been shown to upregulate the expression of intercellular adhesion molecule-1 (ICAM-1), which attracts macrophages that accelerate the formation of precancerous lesions by remodeling the extracellular matrix [32]. Mutant KRAS also induces Krüppel-like factor 4 (KLF4) expression, which subsequently leads to metaplasia and formation of pancreatic intraepithelial neoplasia (PanINs) [33]. Furthermore, oncogenic KRAS mutations can induce constitutive activation of SRY (sex-determining region Y)-box 9 (SOX9), which acts as a cofactor that regulates downstream gene transcription, ultimately leading to cell proliferation and PanIN formation [34].

The mutations in the KRAS gene were found to impair GTPase activity, allowing KRAS to be continuously linked to GTP [35,36]. Thus, the constitutively active KRAS gene is believed to permit uncontrolled cellular proliferation and division and enhance neoplastic invasion and metastasis [37,38,39]. The activating KRAS mutations were observed to be the initial process of pancreatic carcinogenesis in different in vivo studies [40,41]. However, observations from many mouse models highlighted the inability of KRAS mutation solely to result in PDAC, as the development of PDAC was observed in only 7% of the mice; additionally, KRAS mutations were observed in healthy populations, suggesting the involvement of other factors, in addition to KRAS mutation, to induce PC [35,42,43,44,45,46,47,48,49,50,51]. Other mutations associated with the development of PC are TP53, CDKN2A, and SMAD4, all of which are tumor-suppressor genes that can be inactivated later on in the process of PC development and progression [40,41,52,53,54]. CDKN2A and TP53 inactivation result in genetic instability due to removal of cell cycle checkpoints; additionally, the immunosuppressive effects of TP53 inactivation may potentiate PC progression [55,56]. The acquisition of these mutations results in the progression of different PanINs into PC [57,58,59].

### 2.2. Pancreatic Cancer Environmental Factors

PC is a multifactorial disorder, as chronic inflammation, cigarette smoking, obesity, and diabetes mellites have been implicated in its pathogenesis [60]. Multiple studies have proven the causal association between smoking and PC. Among modifiable risk factors of PC, smoking is regarded as the most significant factor [61,62,63]. The associated inflammatory process can potentiate the activity of pancreatic stellate cells (PaSC), enhancing a desmoplastic response that permits PC progression [64,65]. In addition to the immunosuppressive effects of smoking, nicotine has been proven to accentuate tumor growth and survival, potentially by augmenting HGF-MET and AKT-ERKMYC signaling and enhancing K-RAS oncogene activity [66,67]. In addition to nicotine, other carcinogens, including nitrosamines and polycyclic aromatic hydrocarbons, can generate reactive oxygen species that are mitogenic for significant tumor-suppressor genes (e.g., p. 53) [68].

Obesity has significantly increased PC risk and is associated with worse outcomes [69,70]. Several malignancies, including PC, have been connected with the inflammatory state associated with obesity [71]. High levels of adipose-tissue-produced cytokines such as TNF-α, IL-6, leptin, and adiponectin can influence tumor progression, proliferation, and vascular invasion [72]. In addition, insulin resistance and subsequent high glucose levels linked to obesity provide a suitable environment for cancer cell growth and proliferation. Furthermore, high IGF-1 levels due to increased insulin result in PI3K/MAPK/mTOR pathways hyperactivity that subsequently overactivates the RAS/ERK pathway resulting in cellular division, survival, and proliferation [38]. Diabetes mellitus (DM) is associated with PC. In fact, therapeutic interventions with insulin or oral hypoglycemic agents have been associated with decreased risk of PC with improved glucose metabolism [73,74,75]; additionally, higher PaSC activity was observed in patients with DM, which in the setting of hyperinsulinemia potentiates the desmoplastic response and accentuates transforming growth factor beta1 (TGF-β1) signaling contributing to PC development [64,75,76,77,78]. Other contributing factors may include chronic infections (e.g., Helicobacter pylori, and hepatitis B and C viruses), some dietary exposure, or alcohol; however, evidence to suggest their correlation is insufficient [79].

### 2.3. Origins and Precursors of Pancreatic Cancer

**Figure 1:**
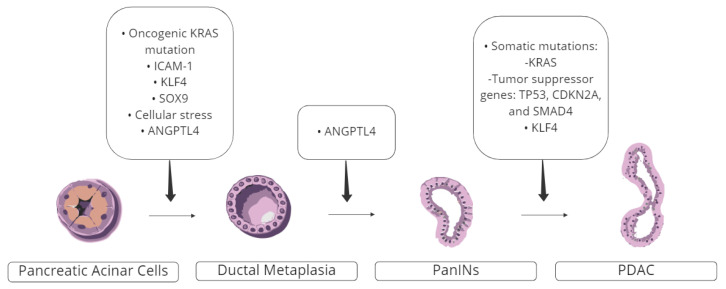
Pathogenesis of Pancreatic Ductal Adenocarcinoma.

Pancreatic ductal adenocarcinoma (PDAC) can arise from different precursor lesions [80]. In the literature, histological subtypes that have been previously highlighted as precursor lesions include intraductal papillary mucinous neoplasm (IPMN), a mucin-producing subtype arising from the pancreatic duct system and mucinous cystic neoplasm (MCN), characterized by the presence of ovarian-type stroma [81]. PanINs comprise the majority of precursor lesions, and the underlying pathological process is illustrated in Figure 1. These lesions are marked by variable epithelial proliferation, and based on the dysplasia degree, they can be classified based on severity into mild (PanIN-1), moderate (PanIN-2), or severe (PanIN-3) [82,83,84]. Although oncogenic KRAS is highly prevalent in PanINs, most of these lesions remain low grade and rarely progress to cancer. Progression is primarily seen with the acquisition of somatic mutations, which accentuate tumor survival and growth. Therefore, most solely KRAS-driven PanINs remain limited to their origin with minimal genomic instability [46,55].

The plasticity of pancreatic acinar cells permits ductal metaplasia during cellular stress, which is believed to give rise to PanINs. Mice observations have found that acinar cells have an extremely high potential to undergo ductal metaplasia [44,85,86]. In addition, based on acinar cell biological function, it is presumed that ductal metaplasia represents a protective adaptation from the acinar digestive enzymes that harm the adjacent ducts [87,88]. As explained earlier, the extracellular matrix of PDAC is desmoplastic, in which many molecules have been shown to potentiate PDAC progression. In a recent study, Angiopoietin-like 4 (ANGPTL4), which is known to upregulate cancer growth and metastasis, was found to accelerate the acinar to ductal metaplasia and eventual progression to PC, supporting the acinar origin of PC [89].

In the presence of cellular stress and/or ANGPTL4, or driven by KRAS-induced ICAM-1, KLF4, and/or SOX9, pancreatic acinar cells undergo ductal metaplasia, which eventually gives rise to PanINs. Acquisition of mutations, mainly affecting KRAS or tumor suppression genes (e.g., TP52, CDKN2A, and SMAD4), accelerates development of pancreatic ductal adenocarcinoma. PDAC: pancreatic ductal adenocarcinoma; ANGPTL4: Angiopoietin-like 4 (ANGPTL4); ICAM-1: intercellular adhesion molecule-1; KLF4: Krüppel-like factor 4; PanIN: pancreatic intraepithelial neoplasia; SOX9: SRY (sex-determining region Y)-box 9.

## 3. Genetic Risk Factors of Pancreatic Cancer

Numerous meta-analyses and pooled studies have been propagated on the etiology of PC. Among the non-modifiable risk factors, ethnicity, family history, and genetics are important factors that predispose to PC progression. Racial differences influence PC progression; for instance, the highest incidence rate was reported in African Americans and the lowest among Asian Americans. Despite the attribution of many modifiable factors to a higher incidence of PC among particular racial groups, other unreported or even unknown factors may be the culprit; this may include, but is not limited to, biological differences in nicotine detoxification and the presence of unrecognized mutant oncogenes that can increase cancer risk [2,90,91]. This was suggested with different expression of TP53 and KRAS genes in a different racial group [92]. Additionally, PC was more prevalent in patients who had a family history of PC; interestingly, Luo et al. reported that 10% of PC cases were observed in the presence of a family history of PC [93]. Although familial PC was typically used to describe cases of PC with two or more first-degree relatives with PC, there is no established consensus on this definition. For instance, the term has also been used to refer to different contexts, including families with three or more relatives with PC regardless of the degree of relationship, when the diagnosis is made in at least one of the relatives prior to the age of 50, as supported by the Brune et al. study that showed an independent risk of PC in families with cases diagnosed earlier than 50 years old [94]. Furthermore, some studies showed a 9-fold and 32-fold increased risk of PC in the presence of a single first-degree relative and three or more first-degree relatives with PC, respectively [95,96]. In the presence of a family history of PC, few genetic mutations were reported in such cases; however, these mutations were not identified in more thorough studies [97,98,99]. For instance, BRCA1/BRCA2 and CDKN2A were positive in up to 17% and 5% of patients with a family history of PC, respectively; however, these observations were seen in a fraction of patients, and further studies are needed to evaluate their contribution [100,101,102,103].

### 3.1. Genetics Role in Pancreatic Cancer

Approximately 10% of patients with PC have a degree of genetic predisposition [104,105]. ATM, APC, BRCA1/2, CDKN2A, MSH2/6, MLH1, PALB2, PMS2, PRSS1, and STK11 were among the most prevalent germline mutations in patients with hereditary PC [96,104]. Additionally, 21 susceptible loci were identified with GWAS analysis. The most commonly investigated loci are summarized in Table 1.

#### 3.1.1. Familial Cancer Syndromes

There is an established association between PC and other familial cancer syndromes. This section illustrates hereditary diseases associated with PC, and the key features of familial cancer syndromes associated with PC are summarized in Table 2.

##### Hereditary Non-Polyposis Colon Cancer (HNPCC)

Mutated mismatch repair (MMR) genes, including MLH1, MSH2/6, or PMS2, have been linked with HNPCC, also known as Lynch syndrome, with an autosomal dominant inheritance. Moreover, colon and endometrial cancer are the most commonly associated malignancies [109,110]. Despite their scarcity, there are a few reports suggesting HNPCC association with PC. For instance, a review of 147 families with mutated-MMR genes showed a PC prevalence of 21% (31 cases). Furthermore, the reported cumulative risk was 3.68% and 5% in patients younger and older than 50, respectively, with an overall 8.6-fold increased risk of PC than non-carrier [111,112]. Among mutated MMR genes, MLH1 and MSH2 were the most frequently associated with PC; however, the data on the actual prevalence are inconclusive. MLH1 carriers had an 8-fold increased risk of PC; however, no risk associated with MSH2, MSH6, and PMS2 was observed [113]. These findings differ from another study, which found a higher incidence of PC in MSH6 carriers than in other MMR mutations [114]. In conclusion, in the presence of a family history of PC, it is plausible that 5 to 10 percent of PC might be attributed to HNPCC.

##### Familial Atypical Multiple Mole Melanoma Syndrome (FAMMM)

Similar to Lynch syndrome, FAMMM syndrome is an autosomal dominant inherited disease. Classically, this condition presents with numerous atypical nevi (>50), which is accompanied by a family history of melanoma in one or more first- or second-degree relatives. A mutated CDKN2A gene on chromosome 9p21.3 that encodes the proteins p14ARF, p16(INK4), p15(INK4b), p18(INK4), and p19ARF is believed to be the culprit. CDKN2A encodes the alternatively spliced tumor suppressors p16INK4a and p14ARF, which function through different signaling pathways. p16INK4a is a cyclin-dependent kinase (CDK) inhibitor that regulates the course of the cell cycle by inhibiting CDK4 and CDK6. In contrast, p14ARF blocks the degradation of the cell-cycle regulator p53 by creating a stable nucleoprotein complex with Mdm2 [115,116,117]. Germline alterations result in an increased risk of early or multiple melanomas and PC FAMMM is associated with a 13- to 22-fold increased risk of developing PC. Further analysis revealed the detrimental mutations in the CDKN2A gene, including p.L65P, p.G67R, c.-201ACTC > CTTT, p.R144C and the founder mutations p.E27X and p.G101W (the “p16-Leiden” mutation), the latter of which was linked with PC cumulative risk of 17% by age 75 in patients of Dutch-decent [156,157,158,159]. Recent research suggests that the p16 protein plays an essential role in pancreatic carcinogenesis. The pathogenic variant affecting p16INK4a encoded protein was positive in 58% of cases among Dutch families with PC. These findings were supported by the reduction/absence of immunohistochemistry expression in up to 80% of PC studied cases [57,118,119,120,121,160,161].

##### Peutz–Jeghers Syndrome (PJS)

As the earlier mentioned syndromes, PJS is a rare autosomal dominant disease characterized by colonic hamartomatous polyps, in addition to oral, buccal, and digital pigmentation. In more than two-thirds of cases, the mutated STK11 (19p13.3) gene is the main culprit, which is a tumor-suppresser gene, that encodes for the Serine/Threonine Kinase 11 (Liver Kinase B1). This protein kinase requires the binding of MO25 and Ste20-related adaptor protein (STRAD) for its activity. Although the exact role of STK11 in PC is not fully known, it is suggested that STK11 controls apoptosis in rapidly proliferating cells (e.g., intestinal epithelium).

It inhibits mTORC1 indirectly by phosphorylating AMPK, which accounts for the opposite response of both pathways to AMP:ATP ratios. In nutrient-deficient environments, ATP depletion causes the ratio to shift toward AMP, thus enhancing AMPK activation. In turn, AMPK phosphorylates TSC1/2 to promote GAP activity toward Rheb, thereby inhibiting mTORC1. However, it was also shown that AMPK suppresses mTORC1 by phosphorylating raptor, the mTORC1 scaffold protein. Therefore, AMPK has the ability to control mTORC1 both directly and indirectly [122,123,124].

Mutant STK11 copy is noted to raise the lifetime risk of cancer by 15 fold compared to the general population and seems to be especially detrimental in women (20 fold) due to an elevated risk of breast and gynecological cancers. PC risk also rises by 11 to 36% in these individuals; nevertheless, up to 30% of PJS patients are caused by a mutation in an undiscovered gene that imparts significant cancer susceptibility [125,126,127,128,129].

##### Hereditary Breast and Ovarian Cancer Syndrome (HBOC)

HBOC is characterized by an increased susceptibility to breast and/or ovarian cancer. Although BRCA1 and BRCA2 represent the majority of associated mutations, other mutations may include TP53, PTEN, CDH1, ATM, CHEK2, or PALB2. These patients are more susceptible to developing PC than the average population. A pooling of 24 studies’ results showed an increased risk of up to 3% and 7% of developing pancreatic cancer in patients with BRCA1 and BRCA2 mutations, respectively [130,131]. Interestingly, patients with BRCA2 mutation appeared to have a higher risk of 17% developing PC with the involvement of three or more relatives [105,132,133]. Although BRCA1 has the notion of being less prevalent in PC, a similar rate to BRCA2 or even higher was reported in patients with an earlier-onset presentation [134,162].

##### Familial Adenomatous Polyposis (FAP)

FAP is characterized by a high number of colonic polyps. This autosomal dominant condition has a nearly 100% penetrance of adenomatous polyps. However, extracolonic manifestations can be variable presentations. Adenomatous polyposis coli (APC) gene is a tumor-suppressor gene on chromosome 5q21-q22 [163,164,165]. APC downregulates the Wnt/β-catenin signaling pathway. Mutated APC has been observed initially in patients with colorectal cancer [166,167,168,169]. In fact, more than 80% of colorectal cancer had an inactivated APC gene, rendering it the most prevalent mutation in colorectal cancer [135]. Additionally, APC has been identified in extra-colonic cancers, including breast, lung, and pancreatic cancer. Furthermore, it has been shown that APC can initiate tumorigenesis in Wnt-independent signaling mechanisms, which also contributes to the development of chemoresistant malignancies, suggesting a tissue-specific effect of APC on carcinogenesis [136,137,138]. The risk of pancreatic cancer increased up to 4 fold in patients with FAP [139,162]. However, the studies in diverse populations are limited, with other studies reporting far lower risk. Therefore, further investigations may be needed to establish a more accurate association [140].

##### Li–Fraumeni Syndrome (LF)

LF syndrome has been associated with an increased risk of malignancies. Almost half of the patients develop cancer by the age of 30. This autosomal dominant syndrome is caused by a germline mutation that involves the TP53 gene, resulting in a broad range of childhood and adult-onset malignancies [141,142] The most commonly observed malignancies in patients with LF are breast, and adrenocortical cancers, in addition to sarcomas and leukemias. Increased risk of lymphoma, melanoma, lung, prostate, and ovarian cancers was also reported. In addition to the increased risk of PC, Aversa et al. reported an association of LF with a primitive neuroectodermal tumor of the pancreas [143,170]. TP53 alterations remain rare, with a prevalence of 0.005–0.01% among the general population [144]. The relative risk of developing PDAC in affected patients was not estimated in a high population level study; however, an analysis of 24 families with LF estimated an approximately 7-fold increase [145,146].

#### 3.1.2. Hereditary Pancreatitis (HP)

The first case of HP was reported initially in 1952. It was noticed after diagnosing repetitive acute pancreatitis in the same family group [147]. However, the autosomal dominant inheritance was confirmed years later by Le Bodic et al. [148].

This condition has a penetrance rate of 80%, and it uniquely presents with recurrent attacks of acute pancreatitis that can subsequently progress into chronic pancreatitis [171,172]. Mutated cationic trypsinogen gene (PRSS1) on 7q35 accounts for 70% of hereditary pancreatitis, which results in impeding of trypsin inactivation, rendering persistence of zymogen activation and auto-digestion of pancreatic tissue [172,173]. The persistent inflammation and tissue damage are the main drivers of PC progression in these patients. Furthermore, smoking has been linked with a 2-fold increased risk of PC in this patient group [149,150,151]. Notably, patients with HP constitute a high-risk group for developing PDAC with an estimated accumulated lifetime risk of up to 50% [152,171]. Given the significantly elevated risk, Scholten et al. emphasized the importance of prophylactic pancreatectomy for this patient population and recommended utilizing shared decision tables to balance the risks in lieu of lifelong surveillance as an alternative management against the benefit of reducing PDAC risk [153].

#### 3.1.3. Cystic Fibrosis

Cystic fibrosis represents the most common genetic disease in Caucasians It is inherited in an autosomal recessive fashion, and it is caused by a mutated cystic fibrosis transmembrane conductance regulator (CFTR) gene that secretes chloride and HCO3- ions across the apical surface of the epithelial cells. The impaired electrolytes secretion and, secondarily, water reabsorption results in an increased mucus viscosity and exaggerated inflammation [154]. PC association with cystic fibrosis has been reported in many studies, including case series of 28,511 cystic fibrosis patients with an OR of 31.5 (95% CI = 4.8–205) for developing PC [155]. Additionally, even CFTR mutation carriers had a higher risk of developing PC compared to non-carrier, which was reported in a large-scale meta-analysis that pooled 1674 PC patients versus 19,036 controls, which confirmed a statistically significant increased risk (OR = 1.41; 95% CI = 1.07–1.84) [174]. Although the pathogenesis behind increased PC risk in patients with cystic fibrosis is not completely understood, it may be linked to persistent chronic inflammation, recurrent pancreatitis, and high acidity of the concentrated pancreatic fluid [175,176]. Other interesting findings were linked to the upregulation of MUC4, a transmembrane glycoprotein that appeared to be upregulated in patients with defective CFTR genes. MUC4 has been linked with increased PC progression due to STAT-1 upregulation [175,177,178].

## 4. Screening of Pancreatic Cancer

More than half of PC cases present with metastasis at the time of diagnosis, and less than 20% present are localized. Initiating screening for an average-risk population may not be associated with improved outcomes; however, detection of PC in its early stages may contribute to reduced mortality in a specific clinical setting [15,18,179,180]. Katz et al. reported a median cancer-specific survival rate of 26 months versus 4.8 months in patients with stage I resectable PC and unresectable PC [181,182,183]. Furthermore, a long-term surveillance study showed that 90% of detected PC in the high-risk population were resectable with an improved short-term outcome and a median 3-year survival [184]. Similarly, another long-term surveillance study, consisting of magnetic resonance cholangiopancreatography and optional endoscopic ultrasound, in high-risk individuals harboring germline CDKN2A mutations demonstrated the benefit of screening in this population. Out of 347 patients, 31 (8.9%) developed PDAC; moreover, 83.3% of cases were resectable at diagnosis which enabled resection in 71% of patients, leading to a 5-year OS rate of 44.1% (95% CI, 27.2 to 71.3). On the other hand, nine patients underwent resection for what proved to be low-grade dysplasia, which highlights the importance of selecting screening modalities with high specificity to avoid unnecessary invasive interventions [180].

The extremely low prevalence of PC decreases the utility of any screening modality; even with highly specific testing, the high rate of false-positive results may lead to unnecessary diagnostic evaluation. In addition to the possible hazards of work-up and increased medical expenditure, the emotional burden can be detrimental for any individual receiving such a diagnosis. Therefore, it is crucial that screening approaches be individualized and targeted based on the risk of PC [185].

### 4.1. High-Risk Group

The identification of high-risk groups is crucial to optimize clinical care, prevent any harmful intervention, and mitigate medical expenditure. Certain patient groups should be targeted, including individuals with ≥5% life-time risk of PC (or 5-fold relative risk) as well as the individuals with a history of familial PC, including ≥2 first-degree relative and >2 relatives, with ≥1 being a first-degree relative [18,94,186,187].

The appropriate age of screening initiation is not well established, neither the screening intervals nor the age of screening cessation. The National Comprehensive Cancer Network (NCCN) recommendations suggest a gene/syndrome-based strategy in identifying the most suitable age for screening, summarized in Table 3 [16].

### 4.2. Screening Modalities

For the early detection of PC, no biomarkers or panels of biomarkers with appropriate diagnostic accuracy have been endorsed yet. In this section, we aim to evaluate the existing PC screening and diagnostic modalities and their potential outcomes, including imaging strategies, pathological examination, serological testing, liquid biopsies, and other emerging advanced diagnostic tools.

#### 4.2.1. Serology

Although serological testing may provide an adjunctive diagnostic method, there is no reliable serological marker for the early recognition of PC. There are, however, few promising tumor biomarkers that are discussed and compared in this review.

##### Carbohydrate Antigen 19-9 (CA19-9)

CA19-9 has been studied extensively as a PC tumor marker. It is approved by the U.S. Food and Drug Administration (FDA) for PC; however, it has been utilized in a broader context to include other gastrointestinal, urological, gynecological, and even pulmonary diseases [188]. However, the low sensitivity and specificity (80% and 75%, respectively) limit its usefulness in clinical practice. In addition, CA19-9 showed a low positive predictive value in both asymptomatic individuals (0.9%) and patients with high suspicion of PC (0.5%) [189,190,191]. Furthermore, the expression of CA19-9 necessitates the presence of 1,4-fucosyltransferase, a product that is absent in the Lewis α-β genotype, that can be seen in 5–10% of Caucasians and in 22% of non-Caucasians [192]. Additionally, the high false-positive rate constitutes another limitation, which can be observed in obstructive biliary disease, pancreatitis, cholangitis, chronic liver diseases, or even healthy individuals [193,194,195] or patients with diabetes [196]. Moreover, CA19-9 levels do not correlate with a worse degree of differentiation in pancreatic neoplasms [192]. Although these factors may limit CA19-9 utility, it carries a prognostic value in estimating the therapeutic response, detecting recurrence, or predicting unresectability; the latter, however, may be variable [197,198,199].

##### CEA, CA125, and CA242

Carcinoembryonic antigen (CEA) is recognized as a tumor marker for colorectal carcinoma. However, recent studies have shown a correlation of CEA with extra-colonic disorders, including gastric, pulmonary, renal, and pancreatic diseases. Interestingly, some of these extra-colonic conditions were associated with a higher mean than colorectal diseases [200]. The studies regarding CEA accuracy are variable. Meng et al. observed no superiority of CEA over CA19-9 when used separately, while a more recent meta-analysis showed a higher sensitivity ratio with CA19-9 compared to CEA [189,201]. CA125 association with PC was observed by Luo et al. when CA125 showed a superior outcome in predicting the resectability of PC compared to CA19-9 [202]. CA242 appeared to have the highest specificity (90%) among the prior tumor markers; however, it has a lower sensitivity [189,203,204].

Using CA19-9, CEA, CA125, and CA242 in combination was associated with 90.4% and 93.8% sensitivity and specificity, respectively, much greater than any single marker; therefore, the suspicion of PC should warrant testing these four tumor markers [203].

##### Immunoglobulin G4 (IgG4)

IgG4 is typically used in the evaluation of autoimmune pancreatitis (AIP), which is associated with PC, as discussed earlier in this review. Despite having a high specificity (89–100%), IgG4 has a relatively lower sensitivity of 72% (95% CI = 0.68–0.76) in discriminating AIP from PC [205,206,207,208]. Four serum biomarkers, including CA19-9, globulin, eosinophil, and hemoglobin, were evaluated by Yan et al. to serve as independent markers that distinguish AIP from PC, showing their combinations identified AIP with 92% and 79% sensitivity and specificity, respectively [209]. The addition of serum hybrid κ/λ antibodies to IgG4 resulted in further increased sensitivity [210]. Some immunogenic membrane antigens were identified (e.g., coiled-coil helix coiled-coil helix domain-containing protein 3). However, further studies are required to work on their association with PC [211].

##### Glycoproteomics

Glycoproteomics has arisen recently as a novel testing utility. The tumor-specific alterations may involve protein glycosylation which may potentially mapped and therefore assessed in the early detection of PC. Aronsson et al. [212] created a panel that included IL.17E, B7.1, and DR6, in addition to CA19-9, to identify PC. These biomarkers showed 100% and 90% sensitivity and specificity, respectively, in a patient with stage 1 PC. Additionally, Liu et al. [213] discovered that levels of 25 isomeric biomarkers substantially changed in PC, which had a 93.5% sensitivity and 90.6% specificity in distinguishing PC from unaffected individuals. These findings can be extremely promising in detecting a novel set of non-invasive testing that can potentially detect PC in the early stages.

##### Lipodomic Profiling

Advances in oncolipidomics research have demonstrated that lipid concentrations change significantly in various cancer types [210]. A three-phase study recently showed that changes in serum lipid concentration can be attributed to PDAC. Reduced levels of very long chain monounsaturated sphingomyelins and ceramides were observed. Interestingly, lipidomic profiling had consistently higher sensitivity than CA 19-9 and CancerSeek, independent of the cancer stage, which is particularly relevant for screening high-risk individuals [211]. Combining both CA 19-9 and lipid profiling enhances specificity and constitutes a promising approach for screening. Nevertheless, follow-up confirmatory and clinical studies are crucial before uptake of this approach into clinical practice.

#### 4.2.2. Liquid Biopsy

Liquid biopsy has been recently identified as a novel utility in detecting circulating tumor markers. These markers can be detected from body fluids including serum, urine, cerebrospinal fluid, or saliva. Specifically in PDAC, driver mutations such as KRAS G12V and G12D mutations have been reported to aid in diagnosis by detecting in DNA collected from pancreatic juice despite pancreatic juice cytology yielding negative results [214]. Circulating tumor DNA (ctDNA) and circulating tumor cells (CTCs) represent the most heavily studied markers; however, other markers may include exosomes and microRNAs (miRNAs) [17].

Liquid biopsy can substitute the conventional “invasive” biopsies. In addition, it can provide genomic analysis that provides a better understanding of drug-resistance in cancer patients [215,216]. Fortunately, these markers are evaluated in an accelerating trend carrying a high potential of heralding a new era of diagnosing PC and other malignancies.

##### CTCs and ctDNA

CTCs are solid tumor cancer cells identified in the peripheral circulation; these markers indicate tumor spread and invasion, as they are typically produced with tumor vascular invasion and angiogenesis [217]. CTCs offer substantial predictive significance for PC patients, as their presence is associated with poorer outcomes (HR = 1.558, 95% CI = 1.238–1.908). In addition, a recent study by Abdelrahim et al. observed a lower recurrence-free survival in patients with early-stage PC who are ctDNA positive. These findings can be suggestive of an additional outcome-predicting value of ctDNA [218]. However, due to their limited and varied sensitivity (ranging from 25% to 100%) in relation to the various stages of PC, several experts question the diagnostic utility of CTCs [218,219,220,221,222]. However, combining CTCs and other biomarkers in a liquid biopsy is anticipated to give a reliable, non-invasive diagnostic approach with appropriate sensitivity. For instance, combining CTCs and glypican-1 (GPC-1)-positive exosome detection exhibited the best diagnostic sensitivity of 100% for resectable PC [223]. Due to the rarity of CTCs in the blood, new procedures and equipment are being created to enhance the diagnostic accuracy of CTCs for cancer. Therefore, enhanced technological and methodological techniques must be specified to illustrate this methodology’s regular application.

##### Cell-Free DNA (cfDNA)

cfDNA is 150 and 200 base pairs that are fragmented from the DNA in the plasma. When these fragments are identified following cellular necrosis of the tumor cells, they are often referred to as ctDNA. In addition, using digital droplet PCR (ddPCR), genomic mutations can be identified. For instance, Kras mutation was detected in the bloodstream, aiding the diagnosis of PC [224]. Furthermore, the detection of ctDNA was associated with a poorer outcome, shorter disease-free survival, and higher recurrence rate following resection, findings that suggest the prognostic role of such markers [17,225,226]. Despite all the promising findings, the ctDNA detection rate in the earlier stages of PC is low (48%) in contrast to advanced cases with a detection rate of (75%) [227]. Another limitation of ctDNA as a freely circulating marker is that the prediction of tumor origin can be challenging.

##### Circulating miRNAs

miRNA are tiny (18–22 nucleotides) non-coding molecules. Once activated, miRNAs are involved in post-transcriptional gene regulation. These molecules can be altered, resulting in oncogenesis [228]. The differential expression of miRNAs amongst malignant versus normal tissues can be implemented to detect various malignancies, including PC. For instance, miR-103 and miR-107 overexpression and miR-155 underexpression were observed to differentiate patients with PC from healthy individuals [229]. In addition, a poorer degree of differentiation was associated with a higher expression of some miRNA, particularly miR-155, which was associated with a 2.6-fold and 7.4-fold overexpression in patients with PanIN-2 and PanIN-3, respectively [230]. Moreover, miR-196b was uniquely expressed in PanIN-3 [231]. However, the detection rate is dependent on tumor burden limiting the usefulness of these markers in patients with smaller lesions.

miRNA-based biomarker panel (including; miR-20a, miR-21, miR-24, miR25, miR99a, miR185, and miR191) utilization in PC was associated with a higher diagnostic accuracy compared to CA19-9 (83.6% versus 56.4%) [232].

In addition, the combination of miRNA panel with CA19-9 was associated with a higher AUC of 0.94 (95% CI = 0.90–0.98, *p* = 0.1) compared with CA19-9 alone with an AUC of 0.90 (95% CI = 0.87–0.94) [230]. In summation, using various biomarkers collectively, including, for example miRNA, CA19-9, and MIC-1, can provide a higher diagnostic accuracy [233]. On the basis of these investigations, miRNAs may become among the most prevalent and promising non-invasive biomarkers; nevertheless, more research in bigger cohorts is necessary to verify the final miRNA panels for future routine clinical applications.

##### Circulating Exosomes

Exosomes are 30 to 150 nm extracellular vehicles (EVs) approximately 30–150 nm in size, composed of a lipid bilayer interleaved with diverse membranous proteins [234]. These molecules are freely circulating in the plasma; in addition, they are produced by all the cells regardless of their benign or malignant nature. Interestingly, EVs or circulating exosomes (crExos) have been studied more recently in the early diagnosis of PC. Although different EV-related molecules were identified, including epidermal growth factor receptor (EGFR) and Mucin 1 (MUC1 or episialin), Glypican-1 (GPC-1) has the highest diagnostic value in recognition of PC [235]. Melo et al. [236] observed a 100% sensitivity and specificity in GPC-1 producing crExos from all patients with PC. Moreover, Kras mutation was solely identified as GPC-1+ crExos, supporting their cancer cell origin [236].

#### 4.2.3. Imaging

Important roles are played by medical imaging in PC screening and early detection, preoperative assessment and staging, differential diagnosis, follow-up, and therapy evaluation [185]. However, there is no standard imaging screening technique in place at this time. The United States Preventive Services Task Force (USPSTF) states that imaging-based approaches such as CT, MRI, and EUS have been evaluated as screening strategies in studies including high-risk individuals with inherited genetic disorders or familial PC [223]. As indicated before, EUS is more effective for screening high-risk patients [224].

The role of imaging in PC has risen tremendously with the new advancements in this field; in addition to the early recognition of PC, imaging is crucial in staging, management planning, postoperative assessment, and recurrence detection [198]. There is no common consensus on the preferred modality for screening. Furthermore, the USPSTF did not endorse any imaging modality for PC screening [237,238]. In this review, we will address different imaging methods that have been studied in PC screening. The attributes and advantages of each modality are outlined in Table 4.

##### Transabdominal Ultrasound (TAUS)

TAUS is used as a part of the initial diagnostic tools in evaluating patients with suspected PC. It has a variable sensitivity (75–89%) and specificity (90–99%), which are influenced by the operator skills, anatomical variation of the pancreas, and body habitus [246]. The use of TAUS for the screening of PC is not promising. In fact, most medical societies do not recognize TAUS as a screening tool [247,248,249]. Tanaka et al. [250] implemented an ultrasonographic approach focusing on the pancreas accompanied by periodic screening that improved the sensitivity of detecting pancreatic cysts from 70.2% to 92.2% [251]. This method was used in Japan [252] due to its feasibility; however, TAUS remains far less sensitive and specific than other imaging modalities, with a diagnostic accuracy of 67.5% compared to 98% with endoscopic ultrasound [239].

##### Endoscopic Ultrasound (EUS)

EUS may represent the most sensitive, specific, and accurate testing in diagnosing and screening for PC. It has a diagnostic accuracy of 98%. Furthermore, a systematic review reported a sensitivity of 91–100% in diagnosing PC [239,253]. EUS was found superior to CT scan and MRI in detecting small pancreatic lesions <2 cm with a sensitivity of 94.4% compared to a 50% sensitivity with CT scan [254,255]. In addition, EUS showed a similar result to CT scan in detecting PC resectability. However, the lack of radiation exposure and the ability to obtain a pathological specimen may tip the balance toward EUS over CT scan [256]. Although not commonly used, the contrast enhancement of EUS has been shown to provide further diagnostic accuracy. These findings were supported by the ability of CE-EUS to identify false-negative EUS results in addition to detecting smaller lesions (<15 mm) [257,258]. Moreover, CE-EUS showed a better performance in differentiating malignant from benign pancreatic lesions with a pooled sensitivity and specificity of 94% (95% CI: 91–95%) and 89% (95% CI: 85–92%), respectively [259]. In a recent randomized clinical trial, the diagnostic accuracy with the use of fine needle aspiration (FNA) through CE-EUS compared to conventional EUS was not statistically significant; however, the sampling was more feasible in CE-EUS, which can be very promising as the accuracy of such testing can be influenced with operator skills and experience [260]. Performing KRAS mutation analysis by digital droplet PCR on EUS-FNA histopathology tissue samples significantly increase the sensitivity of EUS-FNA from 71.4% to 91.6%. Such mutation analysis was found to have superior sensitivity to plasma ctDNA KRAS analysis and CA 19-9 [259]. Real-time elastography (RTE), a novel ultrasound-based technology for measuring tissue elasticity, has been utilized to distinguish malignant from benign tumors in many instances. It has a 94.4% diagnostic accuracy, 93.4% sensitivity, and 100% specificity when combined with EUS-FNA [261].

##### Computed Tomography (CT) Scan

CT scan is the modality of choice for diagnosing PC. Some studies reported diagnostic accuracy of 98% in detecting PC [239]. Multidetector row CT scan has had an excellent performance in evaluating local disease progression, vascular invasion, nodal involvement, and distant metastasis [262]. In addition, a pancreatic protocol CT scan was more efficient in differentiating pancreatic lesions from normal tissue by evaluating the attenuation difference with contrast administration [263,264]. Furthermore, a CT scan detected local fatty changes of the pancreatic tissue in the early stages of PC (stage 0 in 42% and stage I in 41.8% of cases) [239]. CT scan can provide additional values with staging, preoperative evaluation, resectability, and postoperative evaluation; the latter can be enhanced with the use of positron emission tomography scanning [265,266,267]. However, a CT scan appeared to be less sensitive and specific than EUS in detecting smaller pancreatic lesions [268]. It would be reasonable to implement a multimodal approach to detect PC in high-risk patients.

##### Magnetic Resonance Imaging (MRI)

MRI can be used in PC for staging, detecting hepatic micro-metastasis, assessing the systematic effects of PC (e.g., sarcopenia), and evaluating inconclusive CT scan results [269,270,271]. The use of MRI for screening may be challenging, given the high cost and availability. In addition, a recent study showed that EUS had outperformed MRI in detecting solid lesions [272].

## 5. Conclusions

The silent nature of PC only renders most of the cases diagnosable in the advanced or metastatic stages. Early detection is associated with better outcomes; however, given the rarity of this cancer, targeting screening toward high-risk individuals should be recommended. Novel liquid biopsy markers, including ctDNA, may serve as non-invasive prognostic and screening methods. CE-EUS appears to be an effective modality to detect PC, with superior outcomes to CT scan and MRI, and may be utilized for screening in first-degree relatives of patients with PC from familial syndromes, carriers of p16 or BRCA2 mutations with an affected first-degree relative, patients with Peutz–Jeghers and Lynch syndrome, and an affected first-degree relative with PC. The combination of the discussed diagnostic tests should be further evaluated in large-scale studies to assess their role in PC detection.

## Figures and Tables

**Table 1:** Characteristics of Common Loci Implicated in Pancreatic Cancer.

Loci	Function	Mutation Effects	Author
17q24.3	Antitumor RNA Regulates the ubiquitination of PTPN11 (SHP2) with subsequent SRC–ERK oncogenic pathway suppression Induces STAT1-dependent antitumor responses	G > A at rs11655237 in LINC00673 may yield cancer susceptibility, since it inflates cellular PTPN11 levels, hence promoting the proliferation and growth of PDAC cells	Zheng et al. (2016) [106]
13q22.1	DIS3 expression	Altered nuclear RNA processing and decay	Hoskins et al. (2016) [107]
5p15.33	Contains TERT and CLPTM1L Reduces telomerase activity through ZNF148 knockdown	rs36115365 variant alters TERT expression through ZNF148	Fang et al. (2017) [108]

TERT: telomerase reverse transcriptase; CLPTM1L: cleft lip- and palate-associated transmembrane 1-like protein.

**Table 2:** Brief Summary of Familial Cancer Syndromes Associated with Pancreatic Cancer (PC).

Familial Cancer Syndromes	Inheritance Pattern	Hallmark Presentation	Associated Genes	Increased Risk of PC	Other Associated Malignancies	References
Hereditary non-polyposis colon cancer (HNPCC)	Autosomal dominant	NA	Mismatch repair (MMR) genes, including MLH1, MSH2/6, or PMS2	8.6 fold	Colon cancer Endometrial cancer	[107,108,109,110]
Familial atypical multiple mole melanoma syndrome (FAMMM)	Autosomal dominant	Numerous atypical nevi (>50), which is accompanied by a family history of melanoma in one or more first- or second-degree relatives	CDKN2A on chromosome 9p21.3	13 to 22 fold	Melanoma	[111,112,113,114,115,116,117]
Peutz–Jeghers syndrome (PJS)	Autosomal dominant	Colonic hamartomatous polyps, in addition to oral, buccal, and digital pigmentation	STK11 (19p13.3) in up to 70% of cases	PC: 11–36% Any cancer: 15 fold (general population) 20 fold (women)	Lung cancer Gastric cancer Colon cancer Bladder cancer Breast cancer Gynecological cancer	[118,119,120,121,122,123,124,125]
Hereditary breast and ovarian cancer syndrome (HBOC)	Autosomal dominant	NA	BRCA1/BRCA2 (most cases), TP53, PTEN, CDH1, ATM, CHEK2, or PALB2	3–7% and up to 17% if ≥ 3 affected relatives	Breast cancer Ovarian cancer	[126,127,128,129,130,131]
Familial adenomatous polyposis (FAP)	Autosomal dominant	High number of colonic polyps	Adenomatous polyposis coli (APC) gene on chromosome 5q21-q22	Up to 4 fold	Colorectal cancer Breast cancer Lung cancer	[131,132,133,134], [135,136]
Li–Fraumeni syndrome (LF)	Autosomal dominant	NA	TP53	7 fold	Breast cancer Adrenocortical cancer Lung cancer Prostate cancer Ovarian cancer Melanoma Sarcoma Leukemia Lymphoma	[137,138,139,140,141,142,143]
Hereditary pancreatitis (HP)	Autosomal dominant	Repetitive acute pancreatitis that can progress to chronic pancreatitis	Cationic trypsinogen gene (PRSS1) on chromosome 7q35 in 70% of cases	Up to 50% accumulated lifetime risk	None	[144,145,146,147,148]
Cystic fibrosis	Autosomal recessive	Impaired electrolytes secretion and water reabsorption resulting in increased mucus viscosity and exaggerated inflammation	Cystic fibrosis transmembrane conductance regulator (CFTR) leading to MUC4 and STAT-1 upregulation	1.4 fold	Digestive tract cancers	[149,150,151,152,153,154,155]

**Table 3:** Screening Age Recommendations for High-Risk Patient Groups.

Patient Group	Age of Initiation
High-risk genetic mutation, any of: - ATM, BRCA1, BRCA2, MLH1, MSH2, MSH6, PALB2 or TP53 With a first-degree relative diagnosed with PC.	Whichever earlier: - At 50 years old, or - 10 years prior to the first PDAC in the family
Peutz–Jeghers syndrome	At 30–35 years old
Hereditary pancreatitis	- 40 years old, or - 20 years following the onset of pancreatitis
CDKN2A mutation	- 40 years old, or - Within 10 years of the first PDAC in the family

**Table 4:** Summary of Available Imaging Modalities for Screening and Early Detection of Pancreatic Cancer (PC).

Modality	Accuracy	Sensitivity	Specificity	Advantage
TAUS	67.5% [239]	52.4% [240]		Readily available in most health care settings
CT	98.0%	42.8% [240]	64% [241]	Detected local fatty changes of the pancreatic parenchyma
MRI	86.5% [239]	67% [241]		Detecting hepatic micro-metastasis, assessing the systematic effects of PC (e.g., sarcopenia)
EUS	78.6–86.5% [239,242]	83.1–95.2% [240]		The most sensitive, specific, and accurate testing in diagnosing and screening, with a better safety profile given the lack of radio-contrast or radiation exposure
EUS-FNA	85–92% [243]	73.68% [244]	90% [244]	Ability to obtain tissue for histopathologic evaluation from the lesion and from possible regional metastases via fine-needle biopsy (FNB), as well as cyst fluid aspirate for cytology and mutational analysis via fine-needle aspiration (FNA)
CEH-EUS	84.1% [239,242]	94.5% [242]	80% [245]	Provides higher accuracy and technical feasibility to EUS

## Data Availability

Not applicable.
